# β-Glucan as Trained Immunity-Based Adjuvants for Rabies Vaccines in Dogs

**DOI:** 10.3389/fimmu.2020.564497

**Published:** 2020-10-08

**Authors:** Simon Paris, Ludivine Chapat, Nathalie Martin-Cagnon, Pierre-Yves Durand, Lauriane Piney, Carine Cariou, Pierre Bergamo, Jeanne-Marie Bonnet, Hervé Poulet, Ludovic Freyburger, Karelle De Luca

**Affiliations:** ^1^ Boehringer Ingelheim Animal Health, R&D, Lyon, France; ^2^ Université de Lyon, APCSe, Pulmonary and Cardiovascular Agression in Sepsis, VetAgro Sup-Campus Vétérinaire de Lyon, Marcy l’Etoile, France; ^3^ Département Biologie, Faculté des Sciences et Techniques, Université Claude Bernard Lyon 1, Villeurbanne, France

**Keywords:** trained immunity, rabies (canine), beta-glucan, innate immunity, adjuvants, adaptive immunity, Rabisin^®^, trained immunity-based vaccines (TIbV)

## Abstract

The mechanisms of trained immunity have been extensively described *in vitro* and the beneficial effects are starting to be deciphered in *in vivo* settings. Prototypical compounds inducing trained immunity, such as β-glucans, act through epigenetic reprogramming and metabolic changes of innate immune cells. The recent advances in this field have opened new areas for the development of Trained immunity-based adjuvants (TIbAs). In this study, we assessed in dogs the potential immune training effects of β-glucans as well as their capacity to enhance the adaptive immune response of an inactivated rabies vaccine (Rabisin^®^). Injection of β-glucan from *Euglena gracilis* was performed 1 month before vaccination with Rabisin^®^ supplemented or not with the same β-glucan used as adjuvant. Trained innate immunity parameters were assessed during the first month of the trial. The second phase of the study was focused on the ability of β-glucan to enhance adaptive immune responses measured by multiple immunological parameters. B and T-cell specific responses were monitored to evaluate the immunogenicity of the rabies vaccine adjuvanted with β-glucan or not. Our preliminary results support that adjuvantation of Rabisin^®^ vaccine with β-glucan elicit a higher B-lymphocyte immune response, the prevailing factor of protection against rabies. β-glucan also tend to stimulate the T cell response as shown by the cytokine secretion profile of PBMCs re-stimulated *ex vivo.* Our data are providing new insights on the impact of trained immunity on the adaptive immune response to vaccines in dogs. The administration of β-glucan, 1 month before or simultaneously to Rabisin^®^ vaccination give promising results for the generation of new TIbA candidates and their potential to provide increased immunogenicity of specific vaccines.

## Introduction

Host defense against infections relies on the several parts of immunity, composed of two arms: the innate and the adaptive immunity. The concept of adaptive immune memory is dependent on antigen-specific T and B-lymphocytes that are able to recognize a diverse array of pathogens in a highly specific manner, which is the foundation of vaccine functionality. However, the last years have seen an increasing amount of publications describing features of the memory of the innate immunity (1, 2). This memory enables a heightened response to secondary exposure to homologous as well as heterologous pathogens. Among the cells taking part in trained immunity, monocytes have been largely described while some studies support the role of NK cells and dendritic cells (3, 4). It was recently demonstrated that the monocytes can be trained with pathogens (*Candida albicans*) (5), vaccines (BCG) (6), or prototypical agonists like β-glucans initially purified from *C. albicans* (7), to induce deep epigenetic and metabolic modifications (8, 9). This leads to enhanced inflammatory cytokines (TNF-α, IL-6, IL1-β) secretion when the host encounters pathogens mimicked *in vitro* by LPS or *in vivo* in humans by yellow fever attenuated vaccine (10). We confirmed in an *in vitro* model of training of macrophages that the trained innate immunity is also present in other mammals like dogs with cellular mechanisms similar to those described in mice and humans (11).

The description of trained immunity has set new therapeutic goals, which are starting to be investigated in clinical settings (12, 13). A wide range of applications can be found for trained immunity from the use in fish to increase resistance to infection (14), to adjuvant strategies in human cancer therapy (15). Trained immunity-based protection has been theorized and later assessed in mice, against bacterial infections (16), and in humans with a model of yellow fever vaccination (10), both with conclusive results. Combining TI-based protection with vaccine design would require to refine the kind of adjuvants used to improve, polarize and elongate immune response to vaccine antigens (17, 18). Here, we propose the use of β-glucan to serve as a novel kind of adjuvant for trained-immunity based adjuvantation.

In this study, we assessed the potential of β-glucans to induce innate immune training in dogs, as well as their impact on the adaptive immune response to an inactivated rabies vaccine (Rabisin^®^). Injection of β-glucan was performed 1 month before vaccination with Rabisin^®^ supplemented or not with the same β-glucan used as adjuvant, i.e., concomitant to rabies vaccination. For this purpose, we selected a β-glucan extracted from *Euglena gracilis* as we confirmed it was the best inducer of trained innate immunity based on the results from the *in vitro* model that we developed in dogs (11). The selected molecule was administered subcutaneously in dogs and then regular blood sampling were performed to isolate PBMCs. The first objective of this research was the evaluation of β-glucan capacity to train dog monocytes *in vivo* as it was demonstrated in other species (19, 20). The second objective was the demonstration of trained immunity-based adjuvantation. For this purpose, and for ethical reasons, we proposed to investigate the immune response after vaccination rather than infection (10). After Rabisin^®^ vaccination, we monitored the immune response to the vaccine and evaluated if the specific responses were modified by innate training. 

## Materials and Methods

### Study Design and Sampling

**Figure 1:**
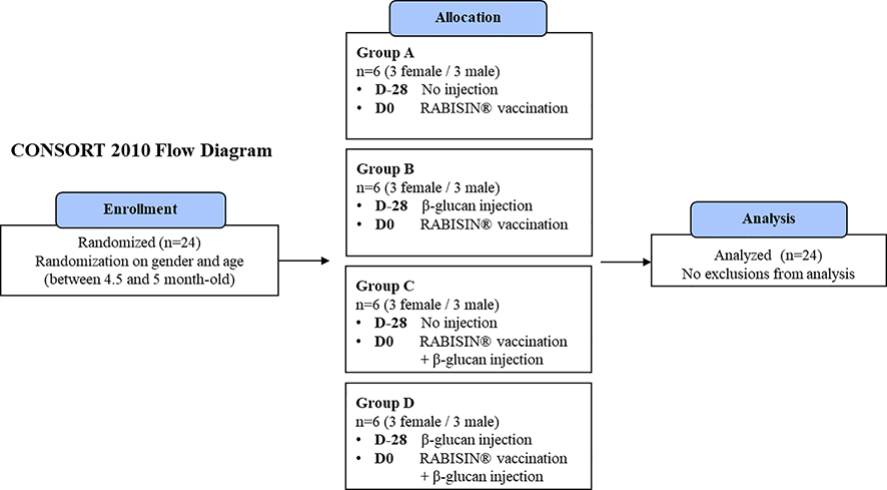
CONSORT 2010 flow diagram of the study design.

Approval of institutional Animal Care and Use Committee (registered in French Ministry of Research as CEEA N°013 was obtained before conducting the study, ensuring that all experiments are conformed to the relevant regulatory standards (directive EU2010/63) and Corporate Policy on Animal Welfare (029-DCPOL-001). Twenty-four conventional Beagle dogs aged between 4.5 months and 5 months were provided by a commercial supplier and were allocated randomly into 4 groups of 6 animals (Groups A to D). The groups were randomized with 3 males and 3 females each. The statistical analysis revealed no difference given the gender of the dogs (**Figure 1**). Age was close between animals of the different groups and had no impact either on the analyses. On day 28, dogs from group B and D received one subcutaneous injection of the preparation of β-glucan in the inter-scapular space. Injected β-glucans show no inflammation at the site of injection and display a very good tolerance. Groups A and B received no injection at D-28. On day 0, dogs from group A and B received one subcutaneous injection of Rabisin^®^ in the inter-scapular space. Dogs from group C and D received one subcutaneous injection of Rabisin^®^ as well as one subcutaneous injection of the preparation of β-glucan less than 2 cm away from the vaccine injection site in the inter-scapular space as summarized in **Table 1**. Blood samples were collected from all puppies on D-28, D-21, D-14, D0, D7, D14, and D28.

**Table 1:** Clinical settings and group repartition.

Groups	D-28 subcutaneous injection		D0 subcutaneous injection	
	Products	Volume (concentration)	Products	Volume (concentration)
**A (n = 6)**			Rabisin^®^	1 ml
**B (n = 6)**	β-glucan	0.5 ml [5mg/ml]	Rabisin^®^	1 ml
**C (n = 6)**			Rabisin^®^β-glucan	1mL - 0.5mL [5mg/mL]
**D (n = 6)**	β-glucan	0.5 ml [5mg/ml]	Rabisin^®^β-glucan	1mL - 0.5mL [5mg/mL]

### Vaccines and Adjuvants

Rabisin^®^ (Boehringer-Ingelheim, Lyon, France) is an inactivated vaccine against rabies adjuvanted with aluminum hydroxide (21), and represents the reference vaccine (Group A). β-(1-3)-glucan from *Euglena gracilis* was purchased from Sigma-Aldrich (Catalog number: 89862; batch: BCBZ6291). 0.5 ml of the suspension was injected at 5 mg/ml, for a dose of 2.5 mg/animal, a dose that was selected according to a review of several clinical studies performed with β-glucan injection (19, 22–25).

### Antibody Response

The antibody response [i.e., serum neutralizing antibodies, total rabies-specific immunoglobulin G concentration (IgG)], concentration of rabies-specific immunoglobulin of IgG1 subclass, and the antibody avidity index) was assessed on D28. Methods used to assess the antibody response are described in Chapat et al. (26).

#### Serum Neutralizing Antibody Titers

Titration of rabies virus neutralizing antibodies (VNAs) was performed by using the fluorescent antibody virus neutralization (FAVN) test according to the technique described by Cliquet et al. (27) with a positive threshold of 0.5I U/ml. Briefly, serial dilutions of sera and a fixed amount of Challenge Virus Standard (CVS) rabies virus (between 50 and 200 TCID50/well) were incubated for 60 min before adding a volume of 50 μl of a suspension of BHK21 cells (4 × 105/ml) to each well. After incubation for 48 h at 37 ± 2°C in a humidified incubator with 5% CO2, the microplates were then fixed in acetone and the cells were labeled by adding an appropriate dilution of a fluorescein isothiocyanate (FITC)-conjugated anti-rabies monoclonal IgG (FUJIREBIO^®^ Diagnostics, Inc. Malvern, Pennsylvania, United States; dilution of 1:133) to each well. Results were assessed by using a microscope equipped for FITC fluorescence. The well was considered positive if one or more fluorescent cells were observed. The 50% endpoint of the antibody content of test sera was calculated by the method of Spearman and Karber. The serum titers were expressed as IU per ml in comparison with the OIE standard serum adjusted to 0.5 IU/ml.

#### Measurement of Total Rabies-Specific Immunoglobulin G (IgG) and Rabies-Specific IgG1 Subclass

The concentrations of total rabies-specific IgG antibodies and rabies specific antibodies of the IgG1 subclass were measured using enzyme-linked immunosorbent assays (ELISAs). Briefly, serial dilutions of sera were incubated with rabies antigen (Boehringer Ingelheim Animal Health, Lyon, France) coated onto the wells of a microtitration plate. The incubation lasted at least 10 h at 5°C. After three washes and a blocking step [5% bovine serum albumin (BSA) in carbonate buffer], peroxidase-conjugated antidog IgG [rabbit anti-dog IgG heavy and light chains (H & L); Nordic Laboratories, Tilburg, The Netherlands] was added in order to detect binding of total IgG. To detect binding of IgG1 subclass, anti-dog IgG1 subclass monoclonal antibodies were added and subsequently revealed using a peroxidase-conjugated F(ab’)2 rabbit anti-mouse IgG (H & L) antibody (Rockland Antibodies & assays, Gilbertsville, PA, USA). Secondary and tertiary reagents were incubated for 1 h at 37°C. Finally, substrate (tetra methyl benzidine) was added to each well and after an incubation period of 30 min at 21°C, the absorbance in each well was quantified by spectrophotometry. The relative titers of antibody in positive control and test sera were calculated by regression compared with in house control serum and were expressed as Log_10_OD_50_. The positive control sera for anti-IgG1 and anti-total IgG ELISA were a pool of sera from animals vaccinated with a double dose of RABISIN^®^ vaccine.

#### Avidity Index

The technique for measuring antibody avidity was based on the detection of anti-rabies total IgG as described above with the inclusion of a dissociation step involving the addition of thiocyanate solution before adding the secondary peroxidase-conjugated anti-dog IgG antibody. In a control experiment, immobilized rabies virus was first exposed to this chaotropic agent to define the concentration and to rule out the possibility that the chaotropic treatment directly stripped the antigen from the ELISA plates. After a washing step, the thiocyanate solution at 0.7 M was added for 14 min at room temperature (RT) in the dark. The effect of thiocyanate enables separation of antigen–antibody complexes with the lowest antibody binding avidity, while preserving the complexes with greatest antibody avidity. The ratio of areas under the curves obtained in anti-rabies total IgG antibody ELISAs performed with or without dissociation gave the avidity index which was proportional to the strength of binding (28, 29). The highest avidity was close to 1, whereas the lowest avidity was close to 0.5–0.6.

### Cellular Responses

Methods used to assess the cellular responses are described in Bommier et al. (30).

#### Isolation of Immune Cells

Canine peripheral blood mononuclear cells (PBMCs) were extracted from whole blood by density-gradient centrifugation over human Pancoll (density 1.077 g/ml, PAN Biotech, D. Dutscher, Issy-les- Moulineaux, France).

For the isolation of monocytes, after extraction from whole blood, PBMCs were seeded at a concentration of 2 × 10^6^cells/well (6.7 × 10^6^cells/cm²) of 96-well culture microplates (CORNING, 353072, Falcon^®^ 96-well Clear Flat Bottom TC-treated Culture Microplate). Cells were incubated at 37± 2°C in a humidified incubator with 5% CO_2_ for 2 h. Adherent monocytes were selected by washing out non-adherent cells with warm PBS.

#### Enzyme-Linked ImmunoSpot (ELIspot) Assays

Rabies specific circulating plasma cells were detected and enumerated by rabies antigen-specific ELIspot assay in blood samples collected respectively on D0 and D7. Briefly, rabies-specific antibody secreting circulating plasma cells were quantified directly within the PBMCs population by rabies specific ELISpot assay. Briefly, PBMCs were added to multiscreen HTS HA ELIspot plates (Merck Millipore, Molsheim, France) coated with canine rabies virus or feline Calicivirus (FCV) antigens (FCV was used as an irrelevant control; Boehringer Ingelheim, Lyon, France) for 24 h. Canine IgG were detected with biotinylated goat anti-dog IgG and horseradish peroxidase-conjugated streptavidin. The spots were counted with a CCD camera system driven by SPOT software (Microvision Instruments, Lisses, France).

#### Rabies-Specific T-Cell Response

PBMCs extracted from whole blood collected on D14 were stimulated in order to detect rabies-specific T-cell responses induced by vaccination. Briefly, rabies-specific interferon (IFN)-γ-secreting cells (IFN-γ spot-forming cells, SFCs) were quantified by an ELIspot assay (EL781, R&D Systems, Lille, France) after stimulation by rabies antigen or control FCV antigen for 48 h. The rabies antigen used consists in the active ingredient from the Rabisin^®^ vaccine after inactivation and before merthiolate addition, concentrated at 100X by a 30%-PEG precipitation in PBS.

### Cytokine Secretion Measurement

#### Quantification of Cytokines by Enzyme-Linked Immunosorbent Assays (ELISA)

Cytokine levels of Interleukin-1β (IL-1β) were detected using canine DuoSet ELISA kits (R&D Systems, Minneapolis, MN; catalog #DY3747) according to the manufacturer’s instructions. The detection limits were as follows: IL-1β, 7.81–500 pg/ml. The optical densities of the peroxidase product were measured by spectrophotometry using a Synergy 2 microplate reader (Biotek, Winooski, VT) at a wavelength of 450 nm. The concentration of interleukin 1β (IL-1β) in the cell-culture supernatant was measured following 72-h stimulation.

#### Quantification of Cytokines by ProcartaPlex Assays

ProcartaPlex assays use Luminex™ xMAP technology for the multi-analyte detection of secreted proteins. The concentration of multiple canine cytokines (IL-2, IL-6, IL-8, IL-10, TNF-α, IL-12p40, IFN-γ, MCP-1, SCF, β-NGF, VEGFα) released into the cell-culture supernatant was measured using a LUMINEX kit (Affymetrix ProcartaPlex Canine 11-Plex, Ebioscience SAS, Paris, France) according to the manufacturer’s recommendations. Sample fluorescence was read on a Bio-Rad Bioplex 200 System and analyzed using Bioplex Manager 6.1 software (Bio-Rad, Hercules, CA).

### Immuno-Stimulation and Immune Training Experiments

#### Immune Training Experiments

After extraction and seeding, monocytes were stimulated with LPS from E. coli O55:B5 for 24 h or left untreated (negative control) Supernatants were then collected and stored at −80°C for further analysis. Cells were cultured in medium (RPMI 1640 (Life Technologies, Villebon sur Yvette, France) supplemented with 10% irradiated fetal calf serum, 1% penicillin-streptomycin (Life Technologies, Villebon sur Yvette, France) and 0.01% β-2-mercaptoethanol (Sigma-Aldrich, Saint-Quentin-Fallavier, France). Finally, cells and supernatants were harvested for characterization of different immunological parameters such as cytokine secretion and cell surface markers.

### Flow Cytometry

#### Antibodies, Instruments, and Analyses

Cells were analyzed using a fluorescence-activated flow cytometer (BD FACSVerse™, BD Biosciences, San Jose, CA, USA) calibrated with BD FACSuite™ CS&T Research Beads; FC Bead 4c, FC Bead 4c+, and FC Bead Violet Research Kits (respective references 650621, 650625, 650626, 650627, BD Biosciences, San Jose, CA, USA). The distribution of doublets was assessed using a side scatter height (SSC-H) vs. side scatter area (SSC-A) density plot followed by a forward scatter height (FSC-H) vs. forward scatter area (FSC-A) plot. Debris and dead cells were assessed both based on forward and side scatter and with viability dyes LIVE/DEAD™ Fixable Yellow Dead Cell Stain Kit, for 405 nm excitation (L349968; Thermo Fisher Scientific Inc., Waltham, MA, USA) before proceeding with the analysis. Acquired events were recorded using BD FACSuite software and analyzed using FlowJo v10.0.7, LLC software. GraphPad Prism software was used for statistical analysis of compiled flow cytometry data. The antibodies against the following antigens were used: CD14 (APC; M5E2; BD Biosciences), CD11b (purified; CA16.3E10; Bio-Rad) coupled with LYNX Rapid APC-Cy7 Antibody Conjugation Kit, CD80 (V450; 16-10A1; BD Biosciences), MHC-II (FITC; CVS20; Bio-Rad), CD369 (Dectin-1; PerCP/Cyanine5.5; 15E2; BioLegend), Arginase-I (PE; 14D2C43; BioLegend).

### Statistical Analysis

A total of 14 immune parameters were included in the analysis. For each immune response parameter, the equality of means between groups was compared using a type III ANOVA test at 5% α-risk. If the vaccine group effect is statistically significant, a Tukey’s test was performed for pairwise comparisons of means between vaccine groups, with adjustment of p-values for multiple comparisons. The normality assumption of residuals was tested using a Shapiro-Wilk test at 1% α-risk level. If the normality assumption of the ANOVA test was not met, the equality of mean ranks between groups was compared using a Kruskal-Wallis test at 5% α-risk. If the vaccine group effect is statistically significant, a Dunn’s test was performed for pairwise comparisons of mean ranks between vaccine groups, with Bonferroni adjustment of p-values for multiple comparisons. In addition of the parametric ANOVA test, the Kruskal-Wallis test was performed for all immune response parameters. In case of plausible normality assumption, the homoscedasticity assumption of residuals according to the vaccine groups was tested using a Bartlett test at 5% α-risk level. If the homoscedasticity assumption of the ANOVA test was not met, the heteroscedasticity was considered into the model. If the vaccine group effect was statistically significant, a Games-Howell test was performed for pairwise comparisons of means between vaccine groups, with adjustment of p-values for multiple comparisons.

An exploratory factor analysis (EFA) was performed to analyze the covariance structure of the immune response parameters and the age covariate (if treated as a quantitative covariate). The immune response parameters were introduced in the EFA in order to build the factors. The normality of each immune response parameter was checked by Shapiro-Wilk test at 1% α-risk level. When necessary, data transformation [e.g., log10(X), or log10(X + 1) when the measured variables contain some 0 values] was applied to immune parameters, to improve within group normality. As the EFA should produce correlated factors, an Oblimin oblique rotation was applied. The number of factors to retain was determined using two criteria: Velicer’s Minimum Average Partial (MAP) test and parallel analysis. The number of factors was concordant with the most clinically meaningful results. The choice of the final model was determined by the factor structure where the factors captured most of the items in a meaningful way, each factor having at least three items with strong loadings (>0.4), and none of the items having strong loadings on more than one factor. For each built factor, the equality of means between groups was compared using a type III ANOVA test at 5% α-risk. If the vaccine group effect was found statistically significant, a Tukey’s test was performed for pairwise comparisons of means between vaccine groups, with adjustment of p-values for multiple comparisons.

All data manipulations and factor analyses were carried out using R version 3.6.2.

## Results

### Evidence for Trained Immunity-Based Adjuvantation With β-Glucan

Using the data generated in our *in vitro* model, we characterized the β-glucan from *Euglena gracilis* as the best training compound for the immune training of canine macrophages. After subcutaneous administration of β-glucan from *Euglena gracilis* in dogs no local reactions or swelling were observed. The follow-up of immune cells numbers did not show any differences between the groups injected and the control groups (**Supplementary Figures 1–3**). We investigated the innate immune parameters described in the scope of trained immunity. Blood-derived macrophages were isolated from blood of dogs one week after the start of the trial, equivalent to D-21. Dogs were separated in two groups, one control (no injection; groups A and C) and one injected with β-glucan (groups B and D). β-glucan injection is considered as a first stimulation, i.e., priming *in vivo*, and, after one week of resting also *in vivo*, cells were extracted and re-stimulated for 24 h *in vitro* with LPS from *E. coli*, mimicking an immune challenge to resume and complete the protocol of trained immunity. We measured the release of pro-inflammatory cytokines, TNF-α, IL-6, and IL-1β, widely described in the trained cytokinic signature (31).

**Figure 2:**
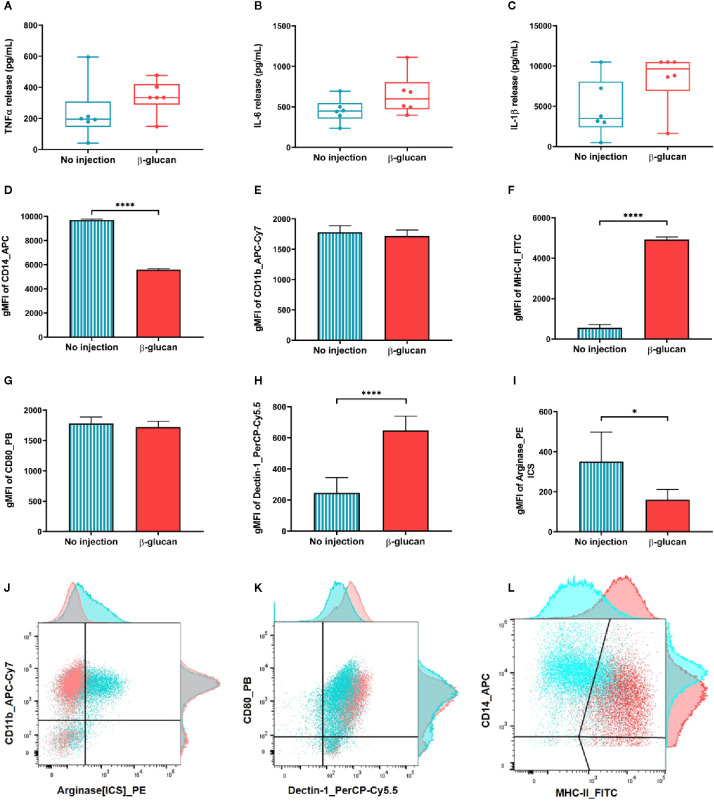
Trained immunity features of macrophages isolated one week after β-glucan injection compared to control. **(A–C)** Cells were stimulated in vitro with LPS for 24 h before quantifying cytokine release in the supernatants. **(D–I)** Immune phenotyping was also performed 24 h post LPS stimulation. **(J–L)** Dot plot illustration of the six markers and their adjunct histograms normalized to mode. *p value < 0.05; ****p value < 0.0001.

TNF-α secretion upon LPS stimulation is slightly increased with a difference between means of 102.0 ± 88.20 (**Figure 2A**). These results do not pass the significance threshold of the p-value due to few individuals driving a high dispersion of the data. IL-6 and IL-1β secretion profiles behave in the same manner with heightened release in the supernatant of the cells isolated from β-glucan-treated dogs (**Figures 2B, C**). The differences between means are respectively of 197.8 ± 120.2 for IL-6 and of 3725 ± 2025 for IL-1β, indicating a global trend toward increase for the three cytokines. No statistically significant differences can be highlighted, the confidence intervals of these results moderately spilling over negative values: [−69.98 to 465.6] for IL-6 and [−787.4 to 8238] for IL-1β. The increase in all three cytokines, though not significant, tends to confirm an implication of trained immunity mechanisms.

Immune phenotyping was performed using flow cytometry to further examine the macrophage phenotype after *in vitro* LPS stimulation. Results are presented in bar charts **Figures 2D–I** and illustrated in dot plots with their adjunct histograms (**Figures 2J–L**). Phenotypic markers CD14 and CD11b were used to discriminate the macrophage population. CD14 expression was found significantly downregulated on the surface of cells from dogs previously injected with β-glucan (p value < 0.0001; **Figure 2D**) while CD11b expression remain comparable between groups (**Figure 2E**). Then, we assessed activation markers associated with pro-inflammatory macrophages, i.e., MHC-II and CD80 (32) as well as arginase, associated to regulatory functions (33). MHC-II expression dramatically increased after LPS stimulation in the group injected *in vivo* with β-glucan (p value < 0.0001; **Figure 2F**). Upregulation of MHC-II implicates an activation of macrophages (34), consistent with the increase in cytokine levels observed. No differences in the CD80 expression were observed between groups (**Figure 2G**). Intra-cellular staining of arginase shows a significant down-regulation of its expression in the cells isolated from dogs treated *in vivo* with β-glucan (p value < 0.01; **Figure 2I**). Arginase downregulation is associated with a switch in arginine metabolism toward an increase of nitric oxide, which is a marker of pro-inflammatory macrophages (9). Finally, the expression of β-glucan receptor Dectin-1 was enhanced in the group treated with β-glucan compared to control (p value < 0.0001; **Figure 2H**). Overall, macrophages isolated from dogs injected one week before with β-glucan showed a more pro-inflammatory profile upon LPS stimulation *in vitro*, with key markers of trained immunity found upregulated compared to the control group.

### β-Glucan Adjuvantation of Rabisin^®^ Tends to Heighten Rabies-Specific Humoral Immune Responses in Vaccinated Dogs

In this study, we used a model of anti-rabies vaccination to characterize the effect of β-glucan to impact the immune response of vaccinated dogs. We used a set of 14 well-characterized immune parameters specific to rabies antigen. Altogether, these parameters help drawing a picture of the efficacy of β-glucan adjuvantation compared to standard rabies vaccination. The multivariate analysis of the different immune parameters, i.e., immune fingerprint, was developed to show a representative image of the immune response to rabies vaccination (26, 30).

The medians and range of 14 immunological parameters assessed in this trial are presented in **Table 2**. Protection to rabies infection is mainly achieved through humoral responses, which were assessed with VNA titers (**Supplementary Figure 4**), a pharmacopeia mandatory test for rabies vaccines, IgG antibody concentrations and their avidity index, IgG1 isotype concentrations, and quantification of specific short-lived IgG-plasma cells.

**Table 2:** Medians and ranges of the 14 immunological parameters included in the exploratory factor analysis, with the addition of VNA titers at D7.

Immune parameters	Day	Indicator	Group A	Group B	Group C	Group D
VNA titers(IU/ml)	D7	Median	0.16	0.72	0.52	0.40
		Range [minimum; maximum]	[0.06; 0.66]	[0.06; 2.62]	[0.29; 3.46]	[0.17; 1.15]
VNA titers(IU/ml)	D28	Median	7.92	12.11	7.50	5.29
		Range [minimum; maximum]	[3.46; 10.45]	[6.01; 41.59]	[4.56; 72.27]	[4.56; 13.77]
Total IgGconcentrations(Log_10_ OD_50_)	D28	Median	2.44	3.18	2.87	2.59
		Range [minimum; maximum]	[2.22; 2.86]	[2.53; 3.61]	[2.49; 3.82]	[2.25; 3.17]
IgG1concentrations(Log_10_ OD_50_)	D28	Median	2.33	3.02	2.62	2.46
		Range [minimum; maximum]	[2.14; 2.81]	[2.52; 3.52]	[2.38; 3.66]	[2.12; 3.11]
Number of IgG-secreting cells/250E3 PBMCs	D7	Median	0.00	13.00	7.00	5.00
		Range [minimum; maximum]	[0.00; 9.00]	[1.00; 26.00]	[0.00; 32.00]	[5.00; 10.00]
Number of IFN-γ-secreting cells/250E3 PBMCs	D14	Median	6.50	2.00	4.50	3.25
		Range [minimum; maximum]	[1.50; 11.00]	[0.00; 8.50]	[1.00; 8.50]	[0.50; 23.00]
IFN-γ release(pg/ml)	D14	Median	37907.68	34282.45	42838.48	28871.47
		Range [minimum; maximum]	[6913.51; 41954.30]	[18670.08; 41089.41]	[27780.47; 50632.98]	[17174.51; 44256.46]
IL-10 release(pg/ml)	D14	Median	3789.61	3945.09	5015.65	2585.72
		Range [minimum; maximum]	[1310.46; 4912.06]	[2277.76; 9633.83]	[2019.95; 7843.86]	[1717.10; 8613.74]
IL-2 release(pg/ml)	D14	Median	443.54	476.93	400.94	380.52
		Range [minimum; maximum]	[397.18; 475.45]	[295.41; 901.93]	[355.40; 695.56]	[276.58; 852.70]
IL-6 release(pg/ml)	D14	Median	798.83	947.07	1062.92	708.50
		Range [minimum; maximum]	[408.38; 920.59]	[471.08; 1743.06]	[571.45; 2616.34]	[451.71; 1640.59]
TNF-α release(pg/ml)	D14	Median	872.02	2015.12	2791.53	855.43
		Range [minimum; maximum]	[129.97; 1528.00]	[617.60; 7465.44]	[682.17; 4419.53]	[255.77; 2314.72]
IL-1β release(pg/ml)	D14	Median	55.94	66.30	60.80	50.90
		Range [minimum; maximum]	[3.52; 90.99]	[3.52; 259.97]	[12.52; 174.09]	[21.86; 80.86]
SCF release(pg/ml)	D14	Median	536.66	864.23	916.98	493.60
		Range [minimum; maximum]	[157.35; 775.92]	[370.80; 1326.26]	[452.33; 1767.22]	[270.69; 1439.44]
β-NGF release(pg/ml)	D14	Median	194.93	209.20	208.48	128.82
		Range [minimum; maximum]	[55.40; 282.51]	[114.01; 1548.47]	[103.93; 637.21]	[64.58; 260.67]
IL12p40 release(pg/ml)	D14	Median	1425.25	1577.44	1774.76	1427.07
		Range [minimum; maximum]	[1154.03; 1735.20]	[699.75; 1835.76]	[1157.24; 2342.68]	[998.80; 2107.06]

**Figure 3:**
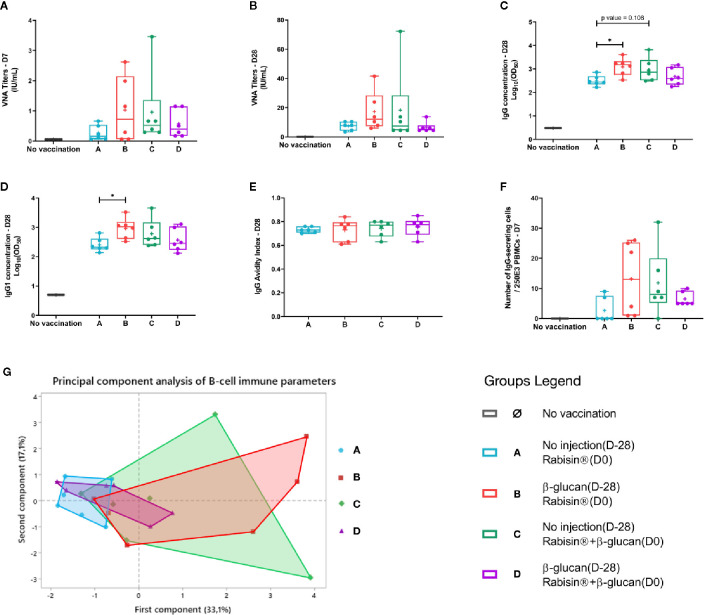
Group comparison of rabies-specific B-cell immune parameters of vaccinated dogs **(A–F)**. Box and whiskers plots of each immunological variables. Kruskal-Wallis tests were performed to compare the values of each immune response parameter between vaccine groups. Dunn pairwise comparisons were performed to evaluate the difference in mean ranks. **(G)** Global image of B-cell immune response using principal component analysis integrating all of the above variables.*p value < 0.05.

Serological parameters, i.e., VNA titers, total IgG and IgG1 concentrations, and avidity indexes were analyzed 28 days after Rabisin^®^ vaccination adjuvanted or not with β-glucan injection. All dogs showed a seroconversion above the protection threshold at 0.5 IU/ml (**Figures 3A, B**). No significant differences in the titers could be highlighted between the four different groups. However, VNA titers observed in group B, β-glucan 1 month prior to Rabisin^®^, showed a substantial higher median (12.11 [6.01; 41.59]) than group A that underwent the standard protocol of Rabisin^®^ vaccination, with one single dose injected at day 0 (7.92 [3.46; 10.45]). The results observed for group A were very similar to the ones in groups C (7.50 [4.56; 72.27]) and D (5.29 [4.56; 13.77]). Dogs from group B showed a significant increase of total IgG concentrations compared to group A (p-value = 0.337; **Figure 3C**). Dogs from group C, who received concomitant injection of β-glucan and Rabisin^®^, followed the same trend of increase without passing the statistical threshold of significance (p-value = 0.108). The double injection of β-glucan in group D, 1 month prior and concomitantly to Rabisin^®^ vaccination, showed no difference compared to group A. IgG1 isotype concentrations displayed a very similar profile to total IgG, with a significant difference between groups A and B (*p-value < 0.05) while such difference was not seen with the two other protocols of β-glucan adjuvantation (**Figure 3D**). No differences between groups were seen for avidity indexes on D28 (**Figure 3E**).

Rabies-specific IgG-secreting cells were quantified at day 7 by ELIspot assay (**Figure 3F**). Only two dogs out of 6 in group A had a detectable number of IgG-secreting cells (0.00 [0.00; 9.00]) whereas all dogs from group B displayed higher results, yet with high and low responders (13.00 [1.00; 26.00]). Dogs in group C showed a similar profile as group B but with higher variability and one non-responder (7.00 [0.00; 32.00]). Finally, IgG-secreting cells quantification of dogs from group D presented a more clustered profile with an intermediate median (5.00 [5.00; 10.00]) between groups B and C and group A. No statistically significant differences were observed between groups. These results are very similar to the ones of VNA titers at day 7. B-cell response parameters were combined in a principal component analysis (**Figure 3G**). This highlights the trend of an enhanced B cell response, with almost no overlap of the response between group A and B, despite the lack of statistical significance partly due to the inter-individual variability.

Overall, B-cell responses, which is the main correlate of protection against rabies (26), display similar profiles between groups A (standard Rabisin^®^ vaccination) and D (double injection of β-glucan). Groups B and C, injected once with β-glucan, 1 month prior or at the same time as Rabisin^®^ vaccination respectively, show a wider dispersion of their response toward an increase of all parameters. However, only total IgG and IgG1 isotype concentrations are able to show a significant difference between groups A and B; all other parameters fail to meet the statistical threshold.

Impact of β-Glucan Adjuvantation of Rabisin^®^ on T-Cell Responses Against Rabies

Cellular responses of T lymphocytes were monitored with quantification of IFN-γ-secreting cells and secretion of several cytokines (IFN-γ, IL-10, IL-2, IL-6, TNF-α, and IL-1β) after canine rabies virus antigen stimulation *in vitro*. IL-2, IFN-γ, and TNF-α are associated with multifunctional T-helper 1 (Th1) response (35). Il-1β reinforce the implementation of Th1 responses by stimulating IL-2 synthesis, which act as a T-cell proliferative cytokine (36). IL-6 is known to be a master regulator of inflammation and has also been described to promote B cell maturation along Th17 responses (37). TNF-α, IL-6, and IL-1β are also well described in the cytokine signature of trained immunity (7). IL-10 plays a role in limiting inflammation and regulating the return to homeostasis (38). Interestingly, IL-10 also promotes B-cell survival and antibody production.

**Figure 4:**
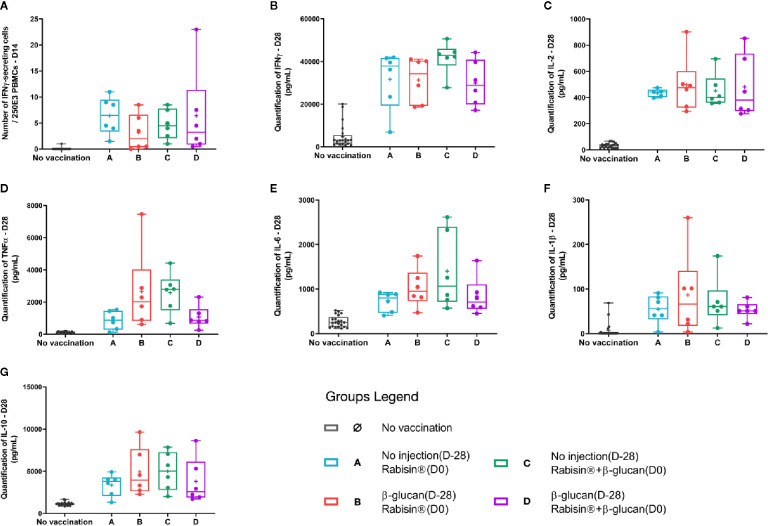
Group comparison of T-cell immune parameters of vaccinated dogs **(A–G)**. Box plots of each immunological variables. Kruskal-Wallis tests were performed to compare the values of each immune response parameter between vaccine groups.

IFN-γ production was assessed using two different techniques, ELIspot and ELISA assays, combining both the number of cells producing this cytokine and the amount. The number of specific IFN-γ-secreting cells was quantified on day 14. Group A showed a higher median (6.50 [1.50; 11.00] SFC/250.10^6^ PBMCs) than the other groups adjuvanted with β-glucan with no statistical differences (**Figure 4A**). Quantification of the IFN-γ secretion showed similar results between groups, with exception for group C, for which, the median was slightly higher, and the response less dispersed but did not show statistical difference (**Figure 4B**).

No differences in IL-2 release following rabies antigen stimulation *in vitro* were observed though groups B, C and D displayed a higher inter-individual heterogeneity (**Figure 4C**). The secretion of TNF-α from groups A and D followed similar results (medians at 872 and 855 pg/ml, respectively) two-times less than the one observed for group B (2015pg/ml) and almost three-times less than group D (2791pg/ml) (**Figure 4D**). A comparable pattern was obtained with IL-6 secretion, for which both groups B and D have at least three individuals driving higher medians and means than groups A and D (**Figure 4E**). Very low IL-1β quantity is detectable in response to rabies-antigen stimulation *in vitro* with no striking differences between groups (**Figure 4F**). IL-10 release behaved in a similar way to TNF-α and IL-6, means of secretion of groups B and C reaching 1.5 times the ones for groups A and D (**Figure 4G**).

### Analysis of the Global Immune Response Against Rabies by a Multivariate Analysis

**Figure 5:**
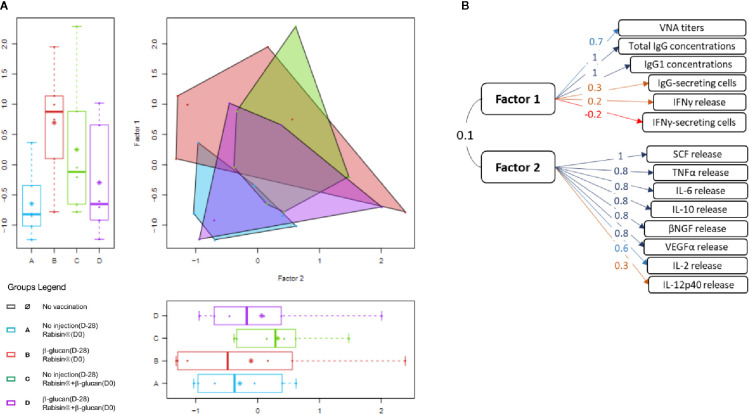
Exploratory factor analysis integrating all immune parameters in the presented study. **(A)** Scatter plot and its adjunct box plots of the exploratory factor analysis recapitulating all parameters. **(B)** Description of the two factors with their coefficients of correlation associated to each variable.

We then investigated all the immune parameters described above with an EFA in order to generate an integrated view of the whole set of data. This unsupervised statistical analysis has been successfully used to study immunological response to Rabisin**^®^** vaccination in dogs (26) and human immune responses to influenza vaccines (39, 40). Every parameter presented in univariate comparison were assimilated to the further described EFA, in a two-factor manner. This model was found best-fitted to the dataset and the separation was relevant with scientific background, each factor recapitulating B- or T-oriented immune response. No correlation was observed between the two factors (coefficient around 0.1). The implication of qualitative covariates (age and gender) was assessed to ensure an unbiased analysis. The representation of the factorial plan is show in **Figure 5A** and the construction of the factors in **Figure 5B**.

The first factor is constituted of all B-related variables described in the first section and also of both IFN-γ-measuring parameters (quantification of secreting cells and of release of IFN-γ) although only VNA titers, total IgG and IgG1 concentrations were highly associated to this factor (coefficient of correlations 1.1 and 0.7, respectively). The second factor was strongly associated with seven variables representing cytokine secretion (IL-2, IL-6, TNF-α, and IL-10) and growth factors secretion (β-NGF, VEGFα, and SCF). The remaining parameters (IL-1β, and IL-12p40 secretion) were poorly associated with this factor (correlation < 0.4). For each factor, there was no statistically significant difference between the groups of vaccination.

The observation of the factorial plan graph highlights the high inter-individual variability, for groups B and C particularly. The area covered by the parameters from group A gather on the lowest scores of both factors with the smallest dispersion. The dataset of group D, showed a very similar profile yet with a larger dispersion. Values of these two groups show really poor discrimination between one another. The immune response of group B was found widely spread in the factorial plan. The factor 1, recapitulating B-cell parameters, exhibited a higher score for this group compared to the reference group A. The scattering of this factor was seemingly attributable to only one individual. The factor 2 was, for its part, very similar in score to the one of group A. Group C also displayed a higher score for the first factor than group A, though not as much as group B. The mean score of the second factor was also higher compared to group A with a noticeable dispersion. However, the statistical significance threshold is not met for any of the two factors in groups B and C compared to group A. Still, the global integrated images of both areas in the factorial plan are from the one of the reference group, indicating a trend in the effect of β-glucan administration.

## Discussion

One of the hurdles in *One Health* is to increase protection to microbial infections both for human and animal species. Due to the variety of circulating pathogens, development of specific vaccines for all of those is not achievable. One way to tackle this issue would be to rely on the non-specific protection that trained immunity seems to offer and for which data still need to be accumulated (16, 41). The features of trained immunity seem promising for this approach with addition of the development of a new area of trained immunity-based adjuvants (TIbAs) (13).

Several analogies are connecting the signature observed in adaptive immunity after vaccination and the results of early innate immunity stimulation by β-glucan injection. Increase in cytokine secretion levels upon a stimulation mimicking an infectious assault is one of the key markers of trained immunity (7). The general trend of increase that we observe here in TNF-α, IL-6, and IL-1β confirms its implementation in dogs *in vivo*. Further analysis of immune phenotyping of macrophages strengthens this hypothesis. MHC-II upregulation in particular, is an activation marker of antigen-presenting cells and associated to increased antigen uptake and processing (42). Such phenotype is widely described in the context of extracellular pathogen but less so for intracellular ones (43). CD80, another activation marker of macrophages along with its functional equivalent CD86, is implicated in initiation and maintenance of CD4+T-cells responses. The expression of CD80 is documented to be elevated in response to LPS (44), which is consistent with the results showed in this study. Yet no differences are shown between groups, which correlates with the equivalent T-cell adaptive response later observed. Arginase, an enzyme transforming arginine in ornithine, is associated with regulatory macrophages (M2-like types) (32). Arginase forms a match with its counterpart iNOS in arginine metabolism, correlated to the anti- or pro-inflammatory features of macrophages (33). Its downregulation in the macrophages coming from β-glucan-treated dogs is another evidence toward trained immunity implementation *in vivo*. Comparable induction of pro-inflammatory profiles of macrophages by immune training has been reported in a context of protection against leptospirosis in hamster, highlighting the clinical use of trained immunity in pathogen-related diseases (24).

The upregulation of Dectin-1 has already been shown to be inducible by pathogens such as fungi containing β-glucans (5). The upregulation of Dectin-1 can also be linked to increase of pro-inflammatory profile. Isoform impact in dogs has not yet been described, A and B isoform in humans are known to have distinct cell surface expression and binding capacities (45). Finally, the low expression of CD14 could be associated with special phenotypes such as non-classical monocytes in humans (46), yet no population has been clearly deciphered in dogs. A fair amount of publications suggest that blood-derived macrophages are not the only populations impacted by an innate training *in vivo* (47), suggesting that bone marrow precursors or resident macrophages at the site of injection would be worth investigating. Taken altogether, these results clearly state a first demonstration of *in vivo* β-glucan’s effects on the immune system of dogs.

The work presented here is consistent with a recent study showing that trained immunity induced by BCG could provide protection in a vaccination model with a live attenuated vaccine against yellow fever (10). With the objective of investigating the role of trained immunity in dogs, we performed our first clinical trial with a vaccine for which we have a good correlate of protection (VNA titers), and well defined immunological parameters to decipher the immune response in dogs. As shown previously, the immune fingerprint of the immune response to rabies vaccines in dogs differs given the adjuvant associated with the rabies antigen (30). Using the multi-parametric factorial analysis, we were able to evaluate adjuvants to decipher the type of response induced.

In this study, we describe the effects of β-glucan injection at several timings on the adaptive immune response to Rabisin^®^ vaccination. Adjuvanted Rabies antigens induce strong Th2 immune responses with Th1 features (48, 49). The results obtained in this trial with the group A confirmed the strong humoral response well described and monitored upon Rabisin^®^ vaccination. The integrated image of the parameters can then be correlated to the vaccine efficacy (26). The timing of β-glucan injection was set to assess two distinct but equally important types of adjuvantation. The trained immunity-based adjuvantation must be distinguished from sole immune-stimulation, used in classic vaccines. The resting period is crucial to decipher those two types. Indeed, β-glucan’s ability to modulate innate responses lies in the description of mechanisms that need time to implement within the cells (metabolic and epigenetic modifications). On the contrary, effects of classic immune-stimulatory compounds as adjuvants are not expected to last several weeks. On the other end of the spectrum, the duration of *in vivo* training effect of β-glucan are still controversial and may depend on the molecules and species (19, 23). Thus, the interval between the first injection, i.e., priming, and the vaccination should be reasonably determined in the same manner as for prime/boost strategies of vaccination. Following the protocol published by Arts et al. (10), we chose an injection schedule 1 month before the vaccination to differentiate between these two adjuvantation methods. Group B, injected with β-glucan 1 month before vaccination, represents the trained-immunity based adjuvantation while group C, injected concomitantly with β-glucan and Rabisin^®^ vaccine, exemplifies a classic way of adjuvantation. Group D collating both injections was used to evaluate of a potential synergistic effect.

Injection of β-glucan 1 month before vaccination showed a consistent increase in all B-cell immune parameters, strongly correlated to protection in rabies. The total rabies-specific IgG antibodies and rabies specific antibodies of the IgG1 subclass are significantly higher than the reference group Rabisin^®^ alone. The other immunological parameters associated with the B cell responses (VNA titers and rabies-specific plasma cells) follow the same trend, with much higher response for group B compared to the control group A.

Interestingly, TNF-α, IL-6, and IL-10 secretions are elevated in response to rabies antigen when β-glucan was injected prior to vaccination. TNF-α is described to modulate adaptive responses by inducing co-stimulatory signals important for both humoral and cellular response maturation (35, 50). IL-6 is required to induce physiological Th1 and Th17 responses and helps to block Treg suppressive effects in synergy with IL-1β (37, 51). IL-10 allows regulation of pro-inflammatory features and help mitigate potential negative impacts (38). Thus, its elevated secretion could show another beneficial effect of β-glucan adjuvantation preventing a deleterious surge in pro-inflammatory mediators. Altogether, these results indicate a heightened immune response to rabies vaccination upon trained immunity-based adjuvantation. This kind of mid-term effects associated with trained immunity-based protection has been documented in humans against yellow fever vaccination (10) and in fish immunity with similar time intervals (19).

The integrated analysis (EFA) distinguishes two factors, one related to B-cell response and one more oriented to T-cell immune parameters. The p-values of both factors are found non-significant, though the first factor is closed to the significance threshold, confirming the results above. The graphical representation clearly shows that groups B and C have an overall increased immune response compared to the reference group A. The underlying reasons of the differences between groups B and C seem to be a stronger Th2 response in group B, shown by factor one, whereas factor 2 increase, Th1-oriented, is observed in group C. It suggests that β-glucan injection 1 month before vaccination tends to increase the immune response without changing its orientation while the concomitant injection does not allow as much elevated B-cell immune parameters but is more effective in increasing T-cell ones. Indeed, simultaneous injection of β-glucan and Rabisin^®^ in group C displays a very similar profile to group B, without passing statistical threshold in the immune parameters associated with humoral response. The implications of these observations can be linked to the necessary interval between the implementation of β-glucan induced trained immunity and its ability to enhance adaptive immune responses (52). Interestingly, the lower values for both B- and T-cell immune parameters are consistently attributed to the same individuals in every group. This clearly favors the hypothesis of non- or low responders to trained immunity. Not only does this increase greatly the dispersion of values but it also reduces the power of the statistical analysis potentially preventing more conclusive results.

Interestingly, the double injection of β-glucan (group D) seems to have a negative impact on the immune response. All parameters measured are diminished in group D compared to groups B and C. Interactions of β-glucan with the antigen uptake and processing are monitored in group C as well, thus the results of group D cannot be attributed to this phenomenon. The most logical cause is a form of immune-modulation due to repeated injections. One hypothesis is the presence of antibodies against β-glucan. Indeed, it has been shown that human serum present high levels of antibodies against several complex sugars from fungal pathogens (53). Although immunoglobulins specific of mannans have been more described, it is now known that anti-β-glucan antibodies are also found (54). The measurement of anti-β-glucan antibodies titers in sera of dogs could be of interest to confirm or disprove their role in trained immunity-based adjuvantation. Cytokine secretion by macrophages after one week of β-glucan injection reflects the early impact of trained immunity. The early results of trained immunity are consistent with the vaccination response of the individuals regarding the amplitude of these responses. Non-responders to BCG vaccines have been described, with insights on discrimination upon changes in DNA methylation (55).

One could consider that the choice of a very potent Rabies vaccine might have limited the full investigation for beta-glucan as a trained-immunity based adjuvant. Rabisin^®^ being a very good vaccine, it is challenging to try to improve it, and even harder to reach statistical significant differences in regards of the immune responses. Furthermore, this vaccine was described to provide non-specific effects in a field study (56). Another challenging choice was made regarding the time frame between the first injection of the β-glucan and vaccination. Although long-lasting effects (from 1 month to several years) were proven to be achievable with attenuated agents like BCG (57). The duration of trained immunity with inert molecules such as β-glucan is still being investigated and discussed in the literature (23). Further studies should aim at evaluating the duration of response *in vivo* with the help of biomarkers of trained immunity in dogs. The concomitant administration to vaccine antigens seems promising nonetheless, with the additional advantage of being convenient for vaccination schedules. Follow-up studies should focus on the trained-immunity based adjuvant role of β-glucan in a context of non-formulated antigens.

In conclusion, β-glucan injection displays a trend of trained immunity-based adjuvantation, theorized from several sources of evidence in this field (1, 12, 13). The present study shows that macrophages presenting an orientation toward a pro-inflammatory profile one week after *in vivo* stimulation are associated with later increased response to Rabisin^®^ vaccination. The link between repolarization of innate immunity modifying, in turn, adaptive immune cells with the use of β-glucan was already documented in the context of tumor-associated macrophages (58). Still the presented work is the first demonstration of such phenomenon in dogs and in a vaccination protocol. This study provides insights for new designs of further investigations with other vaccines candidates, and non-specific protection against infectious agents. Furthermore, this first study in dogs about trained immunity based-adjuvantation gives new insight for the development of adjuvant candidates.

## Data Availability Statement

All datasets presented in this study are included in the article/**Supplementary Material**.

## Ethics Statement

The animal study was reviewed and approved by Approval of institutional Animal Care and Use Committee (registered in French Ministry of Research as CEEA N°013 was obtained before conducting the study, ensuring that all experiments are conformed to the relevant regulatory standards (directive EU2010/63) and Corporate Policy on Animal Welfare (029-DCPOL-001).

## Author Contributions

SP, PB, LF, HP, and KL designed the clinical trial and the associated research. PB conducted the clinical trial. NM-C and CC delivered all the ELISA tests in Boehringer Ingelheim Clinical Analysis Laboratory and handled the interactions with LD31 in charge of the seroneutralization test. SP, LC, P-YD and LP performed the *ex vivo* experiments and read outs. SP and KL analyzed data and wrote the manuscript. All authors contributed to the article and approved the submitted version.

## Conflict of Interest

The authors declare that the research was conducted in the absence of any commercial or financial relationships that could be construed as a potential conflict of interest.
