# Supplementary material for: β-Glucan as Trained Immunity-Based Adjuvants for Rabies Vaccines in Dogs
Source: Front Immunol. 2020 Oct 8;11:564497. doi: 10.3389/fimmu.2020.564497 (PMC7580252; doi:10.3389/fimmu.2020.564497)
Supplement: Supplementary file 1 [file Table_1.docx]

β-glucan as trained immunity-based adjuvants for rabies vaccines in dogs

Supplementary Material

## Supplementary Figures


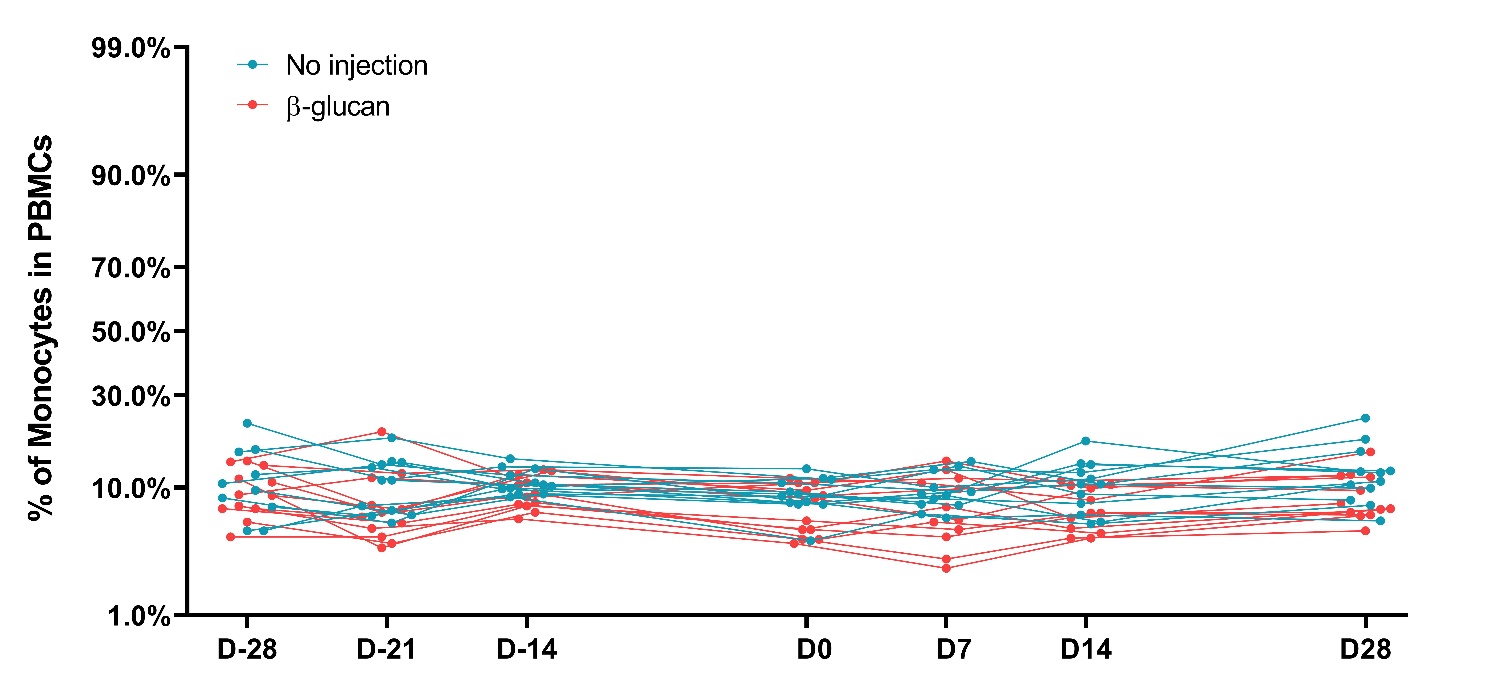


Supplementary Figure 1: Individual Kinetics of monocytes percentage amongst PBMCs in whole blood throughout the clinical trial.


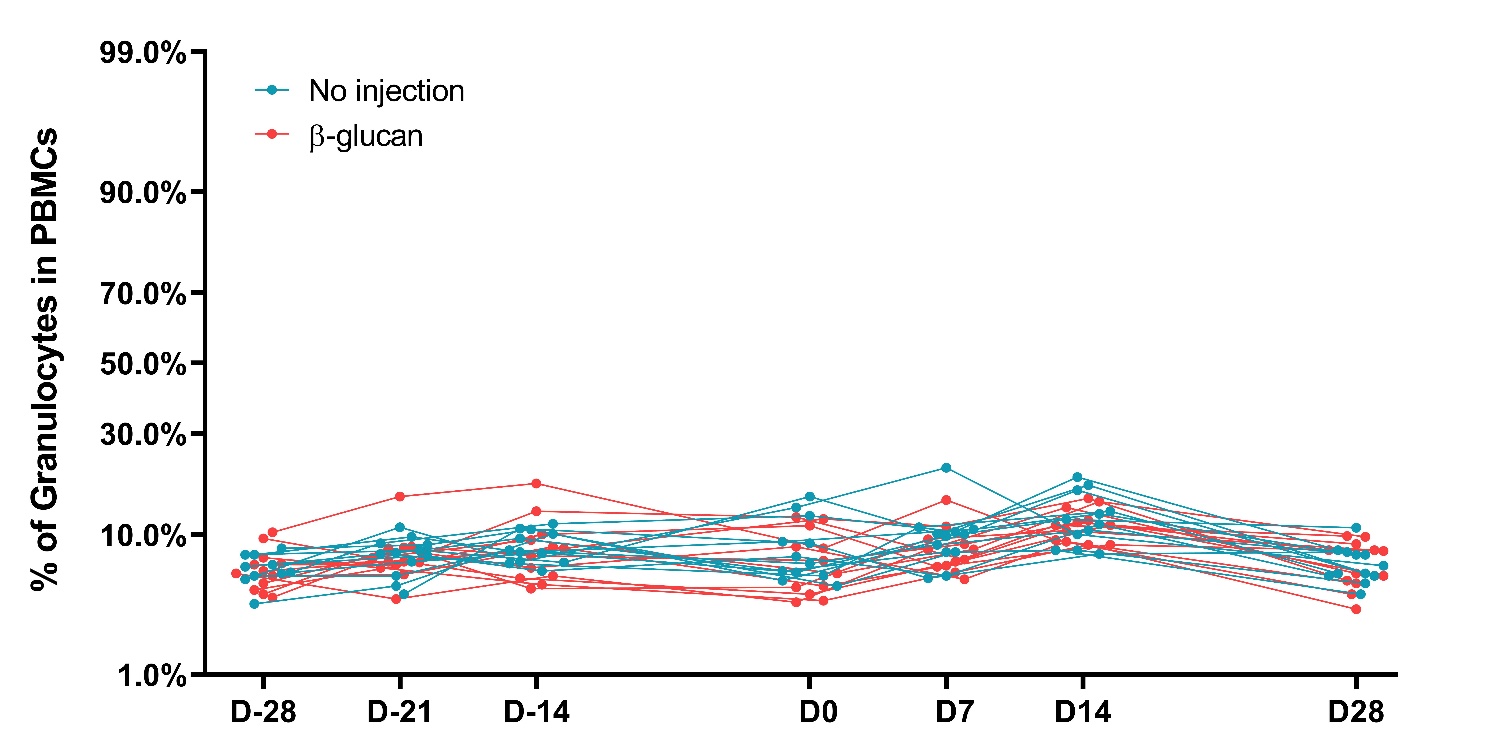

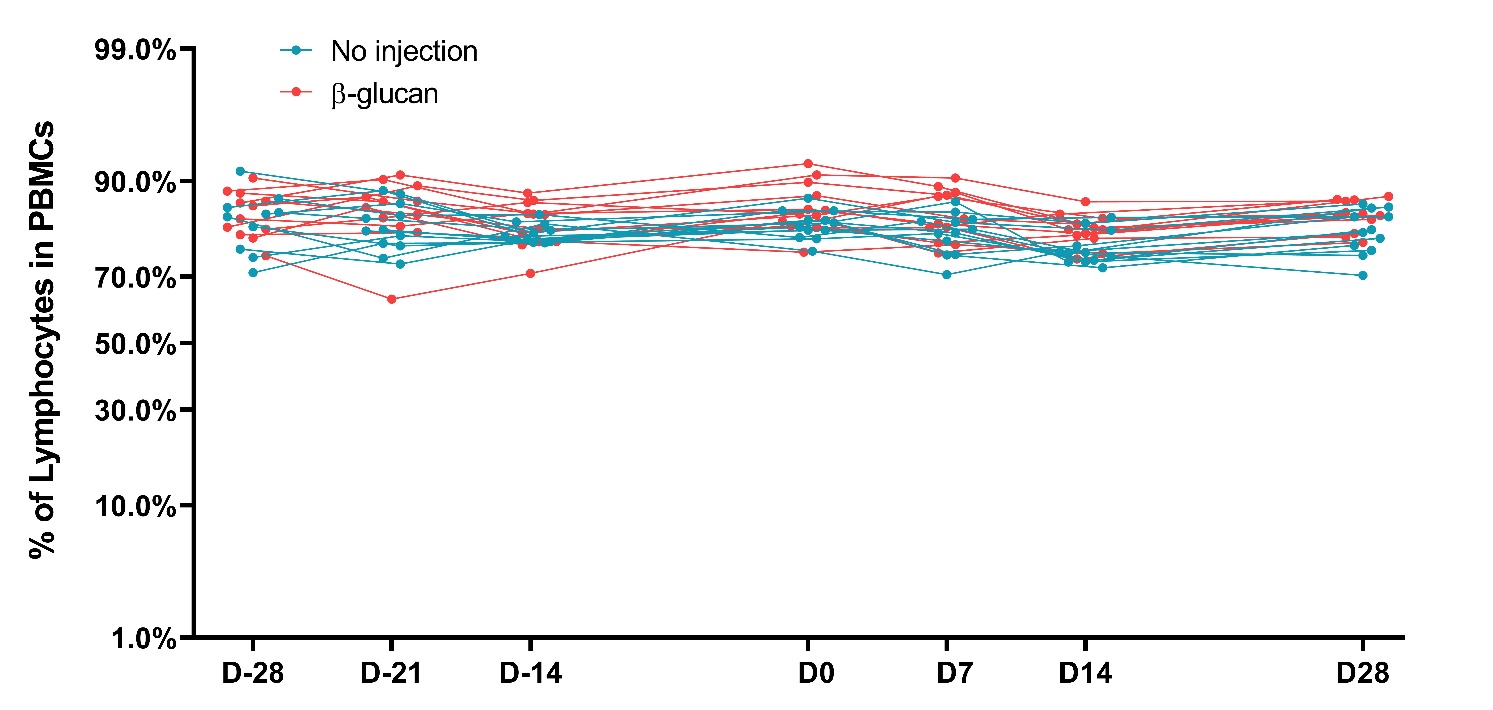


Supplementary Figure 2: Individual Kinetics of granulocytes percentage amongst PBMCs in whole blood throughout the clinical trial.

Supplementary Figure 3: Individual Kinetics of lymphocytes percentage amongst PBMCs in whole blood throughout the clinical trial.

**
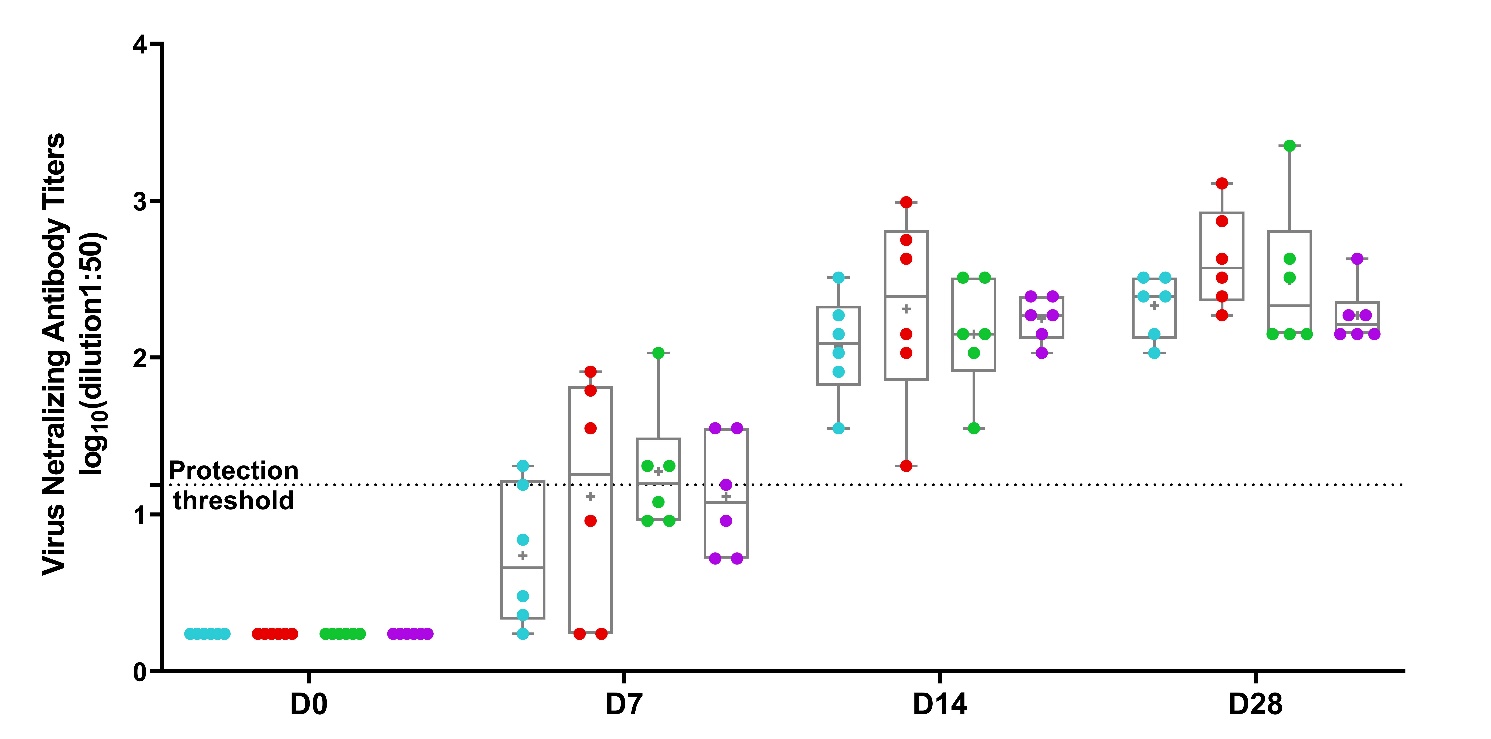
**

Supplementary Figure 4: Individual kinetics of virus neutralizing antibody titers in log_10_(dilution1:50) after vaccination at D0.
